# Preliminary evidence for preserved synaptic density in late-life depression

**DOI:** 10.1038/s41398-024-02837-8

**Published:** 2024-03-14

**Authors:** Thomas Vande Casteele, Maarten Laroy, Margot Van Cauwenberge, Michel Koole, Patrick Dupont, Stefan Sunaert, Jan Van den Stock, Filip Bouckaert, Koen Van Laere, Louise Emsell, Mathieu Vandenbulcke

**Affiliations:** 1 KU Leuven, Leuven Brain Institute, Department of Neurosciences, Neuropsychiatry, B-3000 Leuven, Belgium; 2 Neurology, University Hospitals Leuven, B-3000 Leuven, Belgium; 3 KU Leuven, Leuven Brain Institute, Department of Imaging and Pathology, Nuclear Medicine, B-3000 Leuven, Belgium; 4 KU Leuven, Leuven Brain Institute, Department of Neurosciences, Laboratory for Cognitive Neurology, B-3000 Leuven, Belgium; 5 KU Leuven, Leuven Brain Institute, Department of Imaging and Pathology, Translational MRI, B-3000 Leuven, Belgium; 6 Radiology, University Hospitals Leuven, B-3000 Leuven, Belgium; 7 Geriatric Psychiatry, University Psychiatric Center KU Leuven, B-3000 Leuven, Belgium; 8 Nuclear Medicine, University Hospitals Leuven, B-3000 Leuven, Belgium

**Keywords:** Molecular neuroscience, Depression, Diagnostic markers

## Abstract

Late-life depression has been consistently associated with lower gray matter volume, the origin of which remains largely unexplained. Recent in-vivo PET findings in early-onset depression and Alzheimer’s Disease suggest that synaptic deficits contribute to the pathophysiology of these disorders and may therefore contribute to lower gray matter volume in late-life depression. Here, we investigate synaptic density in vivo for the first time in late-life depression using the synaptic vesicle glycoprotein 2A receptor radioligand ^11^C-UCB-J. We included 24 currently depressed adults with late-life depression (73.0 ± 6.2 years, 16 female, geriatric depression scale = 19.5 ± 6.8) and 36 age- and gender-matched healthy controls (70.4 ± 6.2 years, 21 female, geriatric depression scale = 2.7 ± 2.9) that underwent simultaneous ^11^C-UCB-J positron emission tomography (PET) and 3D T1- and T2-FLAIR weighted magnetic resonance (MR) imaging on a 3-tesla PET-MR scanner. We used analyses of variance to test for ^11^C-UCB-J binding and gray matter volumes differences in regions implicated in depression. The late-life depression group showed a trend in lower gray matter volumes in the hippocampus (*p* = 0.04), mesial temporal (*p* = 0.02) and prefrontal cortex (*p* = 0.02) compared to healthy control group without surviving correction for multiple comparison. However, no group differences in ^11^C-UCB-J binding were found in these regions nor were any associations between ^11^C-UCB-J and depressive symptoms. Our data suggests that, in contrast to Alzheimer’s Disease, lower gray matter volume in late-life depression is not associated with synaptic density changes. From a therapeutic standpoint, preserved synaptic density in late-life depression may be an encouraging finding.

## Introduction

Late-life depression (LLD) is a major depressive disorder (MDD) in later life and has a poorly understood aetiology. Multiple lines of evidence suggest that accelerated biological brain aging might constitute an important pathway to the pathogenesis of LLD. For example, LLD is associated with increased vascular pathology [1], enhanced molecular and senescent changes [2], increased neuroinflammation [3], and lower gray matter volume (GMV) [4]. With regard to the latter, magnetic resonance imaging (MR) studies of late-life depression consistently report lower GMV in brain regions also associated with Alzheimer’s Disease (AD) pathology, such as the frontal [5], temporal [6] and parietal regions [7]. The extent of GMV changes, however, are more pronounced in AD [8, 9]. Importantly, LLD is associated with a twofold risk of developing dementia [10]. Yet the chain of events leading to lower gray matter volume in late-life depression is still unresolved.

Recent research in MDD points towards synapse loss as a mechanism for neural loss [11]. Emerging antidepressant therapies, including ketamine [12], psilocybin [13] and electroconvulsive therapy [14] are thought to enhance synaptic plasticity and have shown promising clinical results in depression [15, 16]. Interestingly, synapse loss is an early feature of mesial temporal atrophy in AD [17, 18], possibly explaining the clinical overlap between LLD and AD [19]. Imaging of synaptic density might therefore shed light on potential neurodegenerative patterns in LLD.

Quantifying synaptic density in humans in vivo recently became possible with the advent of radioligands targeting the synaptic vesicle glycoprotein 2A (SV2A), a ubiquitous presynaptic transmembrane protein [20]. Hence, imaging of SV2A using ^11^C-UCB-J positron emission tomography (PET) provides a suitable proxy for synaptic density in both physiological and pathological conditions [21]. Holmes et al. reported an inverse relationship between depression severity and hippocampal, prefrontal and anterior cingulate ^11^C-UCB-J binding in a group of depressed adults under the age of 60 with and without post-traumatic stress disorder (PTSD) [22]. Whether these findings are generalizable to LLD and whether they might be linked to lower GMV merits elucidation. We therefore conducted a cross-sectional case-control study in older adults that investigated synaptic density and GMV in brain regions implicated in LLD [4]. We hypothesized that GMV and ^11^C-UCB-J binding would be lower in the hippocampus, mesial temporal cortex, anterior cingulate cortex, lateral temporal cortex, prefrontal cortex and parietal cortex in LLD compared to heathy controls, and that synaptic density would be inversely associated with depression severity.

## Materials and Methods

### Participants

All participants from the ongoing Leuven Late Life depression study (see [23] for detailed information regarding sample size and recruitment) enrolled before November 2022 were included. Based on the only ^11^C-UCB-J study investigating differences in depression (MDD not LLD) compared to healthy controls, achieving 80% power at an *α*-level of .008 requires 12 subjects per group. Our LLD group consists of patients over 60 years of age referred to the geriatric psychiatry unit at the University Psychiatric Center (UPC) KU Leuven hospital for unipolar depression as the primary diagnosis, and without any major comorbidity. All fulfilled DSM-5 criteria for major depressive disorder as clinically assessed by a psychiatry resident (TVC) at the time of inclusion. Our healthy control (HC) group consists of 36 age- and gender-matched older adults in the same age range. Exclusion criteria for both groups were screened during a formal medical interview and covered the history or presence of any major disease that may interfere with the investigations, history or presence of a major neurological disorder, history or presence of a psychiatric disease (except depressive episodes for the LLD group), past or current drug or alcohol abuse, and any present contra-indication for PET or MR scanning. The study was approved by the Ethics Committees of the University Hospitals (UZ) Leuven and UPC-KU Leuven (S61968). In accordance with the latest version of the Declaration of Helsinki, all participants provided written informed consent at study entry.

### Clinical assessment and ApoE genotyping

All participants underwent a structured neuropsychiatric interview and neuropsychological testing. We used the Geriatric Depression Scale (GDS, 30 items) [24] and the Montgomery-Asberg Depression Rating Scale (MADRS) [25] to assess depression severity, the Mini-Mental State Examination (MMSE) [26] to assess global cognitive function and the Rey Auditory Verbal Learning Test [27] immediate recall trials (RAVLT-IR) as a proxy for episodic memory. All participants were genotyped to determine apolipoprotein ε (ApoE) status.

### Imaging acquisition and preprocessing

PET and MR brain imaging data were simultaneously acquired on GE Signa 3 T time-of-flight (TOF) PET-MR (GE Healthcare, Milwaukee, WI, USA).

### MR acquisition

High-resolution 3D BRAVO T1-weighted images (plane: oblique; TE: 3.2 ms; TR: 8.5 ms; TI: 450 ms; Flip Angle: 12; slice thickness: 1 mm, voxel size 1x1x1 mm^3^; and 3D T2-weighted FLAIR images (plane: oblique; TE: 137 ms; TR: 8500 ms; TI: 2300 ms; Flip Angle: 12; slice thickness: 1 mm, voxel size 1x1x1 mm) were acquired with a 32-channel head coil on the PET-MR. T1-weighted images were segmented by the default segmentation pipeline of the CAT12 toolbox (v12.6-rc1, Friedrich Schiller University Jena, Jena, Germany) to obtain tissue probability maps. Gray matter volumes-of-interest (VOIs) implicated in LLD (hippocampus, mesial temporal cortex, anterior cingulate cortex, lateral temporal cortex, prefrontal cortex and parietal cortex) were delineated in native space by masking the neuromorphometrics atlas (Neuromorphometrics, Inc) with gray matter probability map thresholded at 0.3. Since we did not expect laterality effects, left and right VOIs were merged into bilateral VOIs. All T1-weighted images were rated as “*good (B)”* by the quality control scale from CAT12, and no abnormalities were observed following visual assessment. Total intracranial volume (TIV, including brain, meninges and cerebrospinal fluid) was estimated by CAT12. White matter (periventricular and deep) lesions (WML) were delineated automatically with an AI based algorithm implemented in icoBrain [28] applied to T1- and T2 FLAIR-weighted images before manual revision by a neurologist.

### PET acquisition

**Fig. 1:**
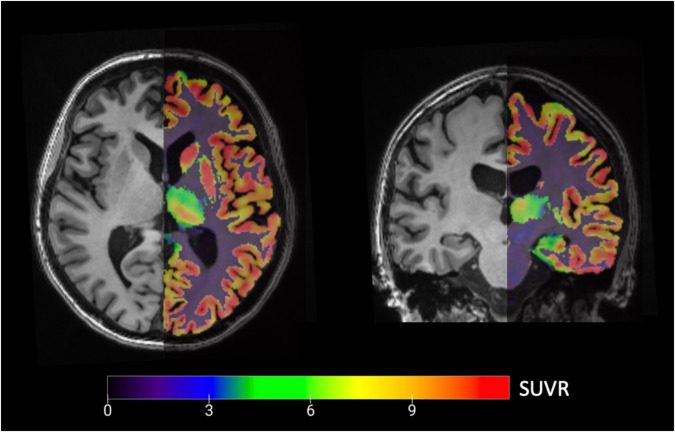
PET-MR imaging. Axial (left, through the prefrontal volume-of-interest) and coronal (right, through the hippocampal volume-of-interest) overlay of a partial volume corrected ^11^C-UCB-J SUVR image (right subparts) registered to the anatomical T1-weighted background for delineation.

^11^C-UCB-J GMP tracer production was done as described previously [18]. On average 182.2 ± 54.4 MBq of ^11^C-UCB-J was injected intravenously 60 minutes before a 30 minute static PET acquisition in list mode. By use of an iterative ordered-subset expectation maximization algorithm (28 subsets, 4 iterations) 6 PET frames of 5 minutes were reconstructed incorporating time of flight data, as well as decay, scatter, and a validated zero echo time (ZTE) attenuation correction [29]. We applied isotropic Gaussian smoothing (4 mm full width at half maximum) to improve signal-to-noise ratio. Off-scanner fully automated all-in processing was done with inhouse scripts (https://github.com/THOMVDC/PSYPET) written in MATLAB (MathWorks, Natick, Mass.) and solely based on SPM12 (v7771, Wellcome Trust Centre for Neuroimaging, University College London) and CAT12 (v12.6-rc1, Friedrich Schiller University Jena, Jena, Germany) to obtain standardized uptake value ratio images (SUVR, Fig. 1). Processing of the 6 PET frames included the following steps: motion correction and averaging of the frames, standardized uptake value (SUV) calculation, and co-registration of the SUV image to the anatomical T1-weighted image. The neuromorphometrics atlas VOI delineation of the anatomical image was projected onto each corresponding SUV image. Since expected GMV differences might induce partial volume effects [30], we corrected images with an validated inhouse region-based voxel-wise partial volume correction (RBV PVC) algorithm (see Mertens et al. [31]. for a detailed description) using the neuromorphometrics VOIs as input regions. The centrum semiovale served as a reference region to calculate SUVR images (see Michiels et al. [32]. for the delineation method) maps after excluding any white matter lesions from the reference region (see MR acquisition for delineation method) [32, 33].

### Statistical analysis

We performed all statistical testing with Python 3.6.8 built-in packages (python.org) as well as SciPy 1.4.1 (scipy.org) and Pingouin (pingouin-stats.org) 0.3.12 packages. Distributions of variables were verified for normality using histograms, q-q plots, and Shapiro-Wilk tests. Mild outliers, defined as beyond 1.5 times the interquartile range (IQR), were included in the imaging analysis. Clinical characteristics were analyzed with descriptive statistics using the chi-squared test (sex), Fisher exact test (apoE e4 carriers, amyloid positivity rate) independent Student t-test (^11^C-UCB-J binding in reference region), or Mann-Whitney U test (all others). To detect VOI-based GMV group differences, we applied analysis of covariance (ANCOVA) with age as a covariate after normalizing the volume of the VOIs by the total intracranial volume (1000‰ x volume_VOI_ / volume_TIV_) [34]. Group differences in ^11^C-UCB-J SUVR values were evaluated with analysis of variance (ANOVA) without age as a covariate since age has no a priori influence on ^11^C-UCB-J binding [35] and there were no significant group differences in age. Partial eta squared was used as a measure of effect size in the group comparisons. Clinical correlations of depression severity, cognitive function and episodic memory with SUVR data in LLD were assessed by Pearson’s r, and clinical correlations with volumetric data in LLD were assessed by Pearson’s r controlling for age. Significant findings were claimed after Bonferroni correction for multiple comparisons (α = 0.008). Given the exploratory nature of our study, we also report findings for a threshold of α = 0.05 as a trend while clearly stating that these are not surviving Bonferroni correction. In addition, we performed post hoc analyses of ^11^C-UCB-J binding differences in (1) late-onset (first episode after 60 years) versus early-onset depression (<60 years) as tested with Mann-Whitney U tests, (2) depression with versus without psychotic features as evaluated with Mann-Whitney U tests, (3) depression with low or high severity of depressed symptoms versus HC based on GDS score (=>20 : severe depression (*n* = 13), <20: mild depression (*n* = 11)), (4) unilateral VOIs in LLD versus HC as tested with ANOVA, (5) LLD versus HC as tested with a voxel-based analysis inside the merged VOIs using SPM12 with a significance level *p*_height, FEW-corrected_ < 0.05 and a cluster extent k_E_ ≥ 50 voxels, (6) LLD versus HC for non-partial volume corrected VOI data (7) LLD versus HC using the reference region without subtracting WML (7) LLD patients taking benzodiazepines vs benzodiazepine naïve LLD patientsLastly (8), a causal mediation analysis was carried with the mediation 4.5.0 package in R 4.0.2 to investigate whether the association between late-life depression and gray matter volume is mediated by synaptic density. Related to this analysis, we also assessed correlations between SUVR and GMV using Pearson’s r, corrected for age.

The experimental procedures (image acquisition and image processing) were performed once and the main statistical analyses were performed three times as a quality assurance check.

## Results

### Demographics and clinical data

We analyzed data from 24 LLD patients (age 73.0 ± 6.2 years, 16 female) and 36 age- and gender-matched healthy controls (age 70.4 ± 6.2 years, 21 female). An additional five healthy controls and ten patients enrolled in the L3D study were excluded from the analysis based on incomplete PET-MR scanning. Within a range of [7–28] on the GDS and a range of [12–48] on the MADRS, depression rating scale scores were significantly higher in the LLD group (*p* < 0.0001) (Table 1); 13 LLD patients scored equal or higher than the moderate-severe depression cutoff (20/30) on the GDS. Eight patients had MDD with psychotic features. At the time of inclusion, all but one LLD patient received antidepressant medication (supplementary table 1) and twelve patients took benzodiazepines. In comparison, no HC took antidepressants. Fifteen patients had at least one early depressive episode (before 60 years), one patient had a previous late-onset episode, and eight patients had never been depressed before the current episode. LLD patients scored significantly lower on the MMSE and RAVLT-IR (*p* < 0.0001). ApoE e4 status did not differ between both groups, the proportion of ApoE e4 carriers was within a normal reference range. PET-MR scanning followed inclusion in a median time of 7 days (IQR = 11) for the LLD group, 35 days (IQR = 119) for the healthy control group.

**Table 1:** Demographical, clinical and imaging characterization.

	Patients with LLD	Healthy controls	Statistic	P-value
Number	24	36		
Age, mean (SD)	73.0 (6.2)	70.4 (6.2)	U = 523	0.17
Female, n (%)	16 (67%)	21 (58 %)	χ2 = 0.14	0.70
Education				0.13
primary education, n	5	3		
secondary education, n	14	15		
higher education, n	4	14		
university, n	1	4		
Geriatric Depression Scale, mean(SD) [range]	19.5 (6.8) [7–28]	2.7 (2.9) [0–12]	U = 855	**<0.01**
Montgomery-Asberg Depression Rating Scale, mean(SD) [range]	26.4 (10.7) [12–48]	0.97 (1.72) [0–7]	U = 864	**<0.01**
MDD with psychotic features, n	8			
MDD without psychotic features, n	16			
Late onset depression, n	9			
Antidepressant medication, n	23			
Previous major depressive episodes, mean(SD)[range]	2.6 (2.6) [0–10]			
Hospitalized at the geriatric psychiatry unit, n	21			
Mini-Mental State Examination, mean (SD)	25.6 (3.1)	28.9 (1.3)	U = 126	**<0.01**
Rey Auditory Verbal Learning Test A - Immediate Recall, mean (SD)	24.0 (10.4)	39.3 (8.7)	U = 659	**<0.01**^**a**^
ApoE e4 carrier, n (%)	3 (12.5%)	6 (17 %)	OR = 0.79	0.09
Framingham risk score	19.8 (13.0)	18.4 (12.5)	U = 332	0.87^b^
Amyloïd positivity rate, n positive / n total tested	1 / 13	4 / 36	OR = 0.66	1^c^
White matter lesions, median mL (IQR)	3.8 (7.7)	2.0 (3.6)	U = 561	0.05
periventricular	6.90 (7.44)	3.21 (2.75)	U = 292	**0.03**
deep	0.66 (0.71)	0.65 (0.71)	U = 363	0.30
11C-UCB-J binding in reference region, mean SUV (SD)	1.1 (0.3)	1.3 (0.3)	T = 2.14	**0.04**
Total intracranial volume, mean mL (SD)	1384 (164)	1372 (148)	U = 441	0.90

*SD* standard deviation, *n* number, *mL* milliliter, *IQR* interquartile range, *SUV* standardized uptake value, *U* Mann–Whitney *U*-value, *OR* odds ratio, *T* Student’s *T* value.

*P* values below 0.05 are indicated in bold.

^a^Listwise deletion of 3 LLD missing data points.

^b^Listwise deletion of 5 LLD missing data points.

^c^Listwise deletion of 11 LLD missing data points. Amyloid status was determined by ^18^F-flutemetamol positron emission tomography.

### Volumetric group differences

**Fig. 2:**
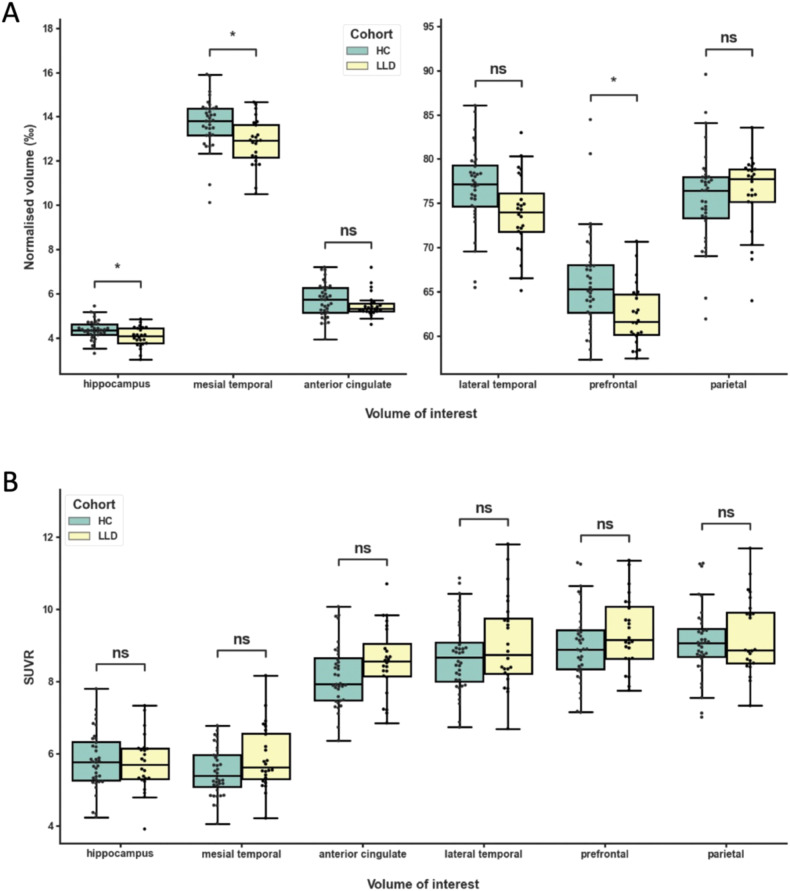
Volumetric and synaptic density group differences. Volume-of-interest (VOI) boxplots of (A) by total intracranial volume (TIV) normalized grey matter volumes (‰) and (B) mean ^11^C-UCB-J SUVRs in the late-life depression (LLD) and healthy control (HC) groups. Boxes represent the interquartile range of the datapoints, whiskers represent the range of the datapoints except for outliers (>1.5 IQR). The range of the Y-axis of plot A is rescaled for the last three VOIs to fit the figure size. The LLD group showed lower normalized grey matter volumes in the hippocampus (*), mesial temporal VOI (*) and prefrontal VOI (*) compared to HC (without surviving Bonferroni correction). The LLD group did not show any differences of ^11^C-UCB-J binding in all VOIs.

We found lower GMV in the LLD group compared to the HC group in the hippocampus (*F*_*(1,57)*_ = 4.56, *p* = 0.04, η_p_^2^ = 0.074), mesial temporal (*F*_*(1,57)*_ = 6.11, *p* = 0.02, η_p_^2^ = 0.097) and prefrontal VOIs (*F*_*(1,57)*_ = 6.33, *p* = 0.02, η_p_^2^ = 0.10), but not in the anterior cingulate (*F*_*(1,57)*_ = 0.55, *p* = 0.46, η_p_^2^ = 0.0095), lateral temporal (*F*_*(1,57)*_ = 4.02, *p* = .05, η_p_^2^ = .066) and parietal VOIs (*F*_*(1,57)*_ = 2.30, *p* = 0.13, η_p_^2^ = 0.039). None of the ANCOVA results survived Bonferroni correction (Fig. 2A, supplementary table 2A).

### Synaptic density group differences

We did not find any differences in ^11^C-UCB-J binding in the LLD group compared to the HC group in the hippocampus (*F*_*(1,58)*_ =0.11, *p* = 0.74, η_p_^2^ = 0.0019), mesial temporal (*F*_*(1,58)*_ = 3.98, *p* = 0.05, η_p_^2^ = 0.064), anterior cingulate (*F*_*(1,58)*_ = 2.99, *p* = 0.09, η_p_^2^ = 0.049), lateral temporal (*F*_*(1,58)*_ = 1.54, *p* = 0.22, η_p_^2^ = 0.026), prefrontal (*F*_*(1,58)*_ = 1.85, *p* = 0.18, η_p_^2^ = 0.031) and parietal VOIs (*F*_*(1,58)*_ =0.33, *p* = 0.56, η_p_^2^ = 0.0057) (Fig. 2B, Supplementary Table 2B).

### Clinical correlations in LLD

#### Volumetry

Within the LLD group, age correlated with GMV in the hippocampus (r = −0.47, *p* = 0.02), mesial temporal (r = −0.42, *p* = 0.04) and parietal VOIs (r = −0.62, *p* = 0.001) but not in the anterior cingulate VOI, lateral temporal VOI and prefrontal VOI (Table 2). Only the association between age and parietal gray matter volume survived Bonferroni correction. Partial correlations correcting for age did reveal a correlation between the parietal GMV and the GDS (r = 0.45, *p* = 0.03), the MADRS (r = 0.44, *p* = 0.03) and the MMSE (r = 0.45, *p* = 0.03) without surviving Bonferroni correction. Prefrontal GMV showed a correlation with the RAVLT-IR (r = 0.56, *p* = 0.01) without surviving Bonferroni correction. No correlations were found between the RAVLT-IR and GMV in the hippocampus, mesial temporal, anterior cingulate, lateral temporal and parietal VOIs after correcting for age. The scatter plots associated with these results can be found in the supplementary materials (Supplementary Figs. 1, 2 and 3).

**Table 2:** Volumetric correlations with LLD clinical outcomes.

	Age	GDS	MADRS	MMSE	RAVLT-IR
VOI	r, P	r, P	r, P	r, P	r, P
Hippocampus	**−0.47, 0.02**	0.21, 0.35	0.28, 0.19	−0.32, 0.13	−0.31, 0.19
Mesotemporal	**−0.42, 0.04**	0.07, 0.76	0.12, 0.60	−0.16, 0.47	0.07, 0.77
Anterior cingulate	−0.17, 0.42	0.24, 0.27	0.33, 0.13	−0.26, 0.23	0.08, 0.73
Lateral temporal	−0.39, 0.06	0.04, 0.87	0.14, 0.52	−0.13, 0.54	0.09, 0.72
Prefrontal	−0.13, 0.55	−0.16, 0.46	−0.06, 0.80	0.28, 0.20	**0.56, 0.01**
Parietal	−0.62, 0.001*	**0.45, 0.03**	**0.44, 0.03**	**−0.45, 0.03**	−0.12, 0.61

*VOI* volume of interest, *GDS* Geriatric Depression Scale, *MADRS* Montgomery–Asberg depression rating scale, *MMSE* Mini-Mental State Examination, *RAVLT-IR* Rey Auditory Verbal Learning Test immediate recall trials, *r* Pearson’s *r*. *P* *P* value.

*P* values below 0.05 are indicated in bold.

*Significant values after Bonferroni correction (*p* < 0.008).

#### Synaptic density

Within the LLD group, age and depression severity did not correlate with ^11^C-UCB-J SUVR in any of the VOIs. A negative correlation was found between ^11^C-UCB-J SUVR and MMSE in the prefrontal (r = −0.48; *p* = 0.02) and the parietal VOI (r = −0.44, *p* = 0.03) without surviving Bonferroni correction, but not in hippocampus, mesial temporal and lateral temporal VOIs. (Table 3) No correlations were found between RAVLT-IR and ^11^C-UCB-J SUVR values in all VOIs.

**Table 3:** 11C-UCB-J SUVR correlations with LLD clinical outcomes.

	Age	GDS	MADRS	MMSE	RAVLT-IR
VOI	r, P	r, P	r, P	r, P	r, P
Hippocampus	0.07, 0.76	0.03, 0.87	−0.03, 0.89	−0.36, .08	−0.19, 0.42
Mesotemporal	0.18, 0.41	−0.09, 0.68	−0.17, 0.43	−0.35, .09	−0.05, 0.82
Anterior cingulate	−0.01, 0.96	0.06, 0.77	−0.12, 0.59	−0.26, .22	−0.11, 0.63
Lateral temporal	0.19, 0.37	−0.07, 0.73	0.10, 0.63	−0.32, .12	<0.01, 0.99
Prefrontal	0.15, 0.48	0.09, 0.66	0.02, 0.92	**−0.48, 0.02**	−0.15, 0.51
Parietal	0.24, 0.25	−0.06, 0.78	−0.05, 0.83	**−0.44, 0.03**	−0.09, 0.71

*VOI* volume of interest, *GDS* Geriatric Depression Scale, *MADRS* Montgomery–Asberg depression rating scale, *MMSE* Mini-Mental State Examination, *RAVLT-IR* Rey Auditory Verbal Learning Test immediate recall trials, *r* Pearson’s *r*, *P* *P* value.

*P* values below 0.05 are indicated in bold. No values survived after Bonferroni correction (*p* < 0.008).

### Post hoc analyses

Post hoc analyses of ^11^C-UCB-J SUVR values in late-onset (after 60 years) versus early onset (before 60 years) depression, and between LLD without versus with psychotic features did not reveal any group differences (supplementary tables 3 and 4). No group differences in ^11^C-UCB-J SUVR were found between low-severity LLD and HC, or between high-severity LLD and HC (supplementary table 5A and 5B). No significant ^11^C-UCB-J clusters were found in the voxel-based analysis inside the merged VOIs. SUVR data not corrected for partial volume effect in LLD vs HC showed similar results to the partial volume corrected data (supplementary table 6). Using the centrum semiovale without subtracting WML as a reference region did neither affect the SUVR results (supplementary table 7 and supplementary figure 4). No significant ^11^C-UCB-J binding differences were found between LLD patients taking benzodiazepines or not (supplementary table 8). Unilateral VOI analysis showed higher ^11^C-UCB-J binding in the left mesial temporal lobe of LLD participants without surviving Bonferroni correction (supplementary table 9). The association between LLD and gray matter volume was not mediated by synaptic density (supplementary table 10A), nor were there any associations between GMV and SUVR in any VOI (supplementary table 10B).

## Discussion

We found no evidence for altered synaptic density in late-life depression as measured by ^11^C-UCB-J in brain regions implicated in depression, while some of the regions showed lower GMV in the LLD group (without surviving Bonferroni correction). Parietal GMV correlated with depressive scales and the MMSE, without surviving Bonferroni correction. Prefrontal GMV correlated with the RAVLT-IR, without surviving Bonferroni correction. VOI-based correlations between ^11^C-UCB-J binding and depressive scales could not establish a link between synaptic density and depression severity, whilst there was some evidence for an inverse correlation with MMSE in the PFC and parietal cortex.

Until recently the hypothesis linking synaptic density with depression had only been investigated ex-vivo in animal stress studies and post-mortem depression studies focusing on the hippocampus and prefrontal cortex [11, 36]. Cell-counting studies incriminated neuronal and glial abnormalities for lower gray matter volume in MDD [37], while histopathological findings showed lower prefrontal synapse-related gene expression hinting at lower synaptic density in MDD [36]. Our finding suggests that there is no regional difference in synaptic density as measured by ^11^C-UCB-J across several cortical regions implicated in late-life depression. This does not preclude alterations in synaptic function since ^11^C-UCB-J might not capture vesicle release dynamics [38]. In contrast, preserved synaptic density might indicate that synaptopathy on a structural level is not a key pathophysiological feature in LLD, and possibly allows reversibility of network dysfunctions. This is in line with the extensive literature indicating a favorable outcome after adequate treatment in LLD [39].

The only other study that has investigated ^11^C-UCB-J in depression focused on unmedicated depressed MDD/PTSD adults under the age of 60 [22]. They reported lower distribution volumes (V_T_, representing specific and unspecific binding) of ^11^C-UCB-J in the depressed group within the hippocampus, anterior cingulate cortex and dorsolateral prefrontal cortex, as well as other regions not assessed in their primary analysis: occipital cortex, parietal cortex, temporal cortex, cerebellum, putamen. There are a number of reasons that make a direct comparison of results challenging. First, the study population differed in terms of age (39.2 ± 12.1 years versus 73.0 ± 6.2 years), diagnosis (MDD/PTSD mixed group versus LLD only), and medication use (unmedicated versus medicated). Aside from clinical heterogeneity, the respective PET quantification methods differed, but were both derived from preclinical validation studies.

GMV in the LLD group was lower in the hippocampus, mesial temporal and prefrontal VOI (none surviving Bonferroni correction). The prefrontal cortex, in concert with the hippocampus, also modulates memory processing in a top-down manner [40]. Episodic memory dysfunction is often reported in late-life depression and correlates well with depression severity [41]. Consistent with the brain aging literature, we found a correlation between age and hippocampal, mesial temporal and parietal gray matter volume (only parietal VOI survived Bonferroni correction).

Gray matter volume decrease is well described in aging [42], but it is not necessarily correlated with a loss of neurons [43] or synaptic density decrease (as measured by ^11^C-UCB-J) [35]. This is in contrast to most neurodegenerative diseases, such as AD where pathology leads to a net loss of synapses [44] and eventually to a loss of neurons and to gray matter volume decrease [17]. In AD the atrophy rate has been estimated to 1-4% a year [45] and has been linked to synaptic density (as measured by ^11^C-UCB-J) decreases even in early stages [46]. If lower GMV in LLD was associated with neurodegenerative-related changes, one would expect lower synaptic density to precede the volumetric changes. In contrast, our findings suggest that even when gray matter volume is lower, net synaptic loss does not exceed net volume loss such that synaptic density remains stable, in a comparable way to age-related gray matter volume loss [35].

Distinguishing etiological and consequential factors that contribute to the clinical presentation of LDD is challenging given the reciprocal relationships between most of these factors. Accordingly, deviation from normative GMV trajectories in LLD [47] might result from or cause LLD in a synergistic manner. The hypothesis of accelerated (brain) aging associated with LLD [48] might explain lower GMV without changes in synaptic density. Age-related GMV decrease might be essentially driven by a reduction in microglia and astrocytes [49]. In addition, cardiovascular and proinflammatory backgrounds that are common in LLD might contribute to GMV reduction without affecting synaptic density [50].

Another (non-exclusive) explanation might be that lower GMV is pre-existing to depression onset. Specifically, lower GMV at baseline does not imply lower density of synapses in that tissue. Lower GMV has been associated with less cognitive, executive and coping reserve towards life stressors [51] and might therefore constitute a vulnerability marker in an aging population.

This is the first prospective study measuring synaptic density in vivo in LLD. As it is challenging to motivate this population for participation in demanding imaging studies, research in the field might be biased towards milder depression. Mild depressive symptoms in later life might be a prodrome of neurodegenerative diseases [52], where synaptic density decrease is increasingly described [53]. Our cohort consisted of patients referred to the geriatric psychiatry unit with a major depressive disorder as the primary diagnosis, and without any major neurological comorbidity. In these terms, preserved ^11^C-UCB-J in late-life depression might be a potential differential diagnostic biomarker between depression as a primary psychiatric disorder and depressive symptoms as a consequence of neurodegenerative disorder.

Some methodological study limitations to the present study are important to highlight. T1-weighted MR segmentation is prone to artefacts and epiphenomena [54], yet the differences in MR measurements we reported in LLD are in line with histopathologic literature and therefore suggestive for GMV differences. We used a simplified quantification method (SUVR) instead of BP_ND_ or DVR as SUVR calculation does not require invasive arterial sampling nor dynamic scanning up to 90 min but only a 30 min scan, thus increasing (clinical) feasibility of the protocol in depression. This quantification method has been validated in healthy aging [33] and in AD [55], but not in LLD. Second, since LLD is often associated with vascular burden, we cannot exclude white matter lesions (WML) having biased the present results. To reduce impact from WML on ^11^C-UCB-J binding in the centrum semiovale (CS), we subtracted WML from the CS masks (see PET acquisition, supplementary table 11). Using the CS without correction for WML as a reference region did not affect the main outcome; no group differences in mean SUVR values were found in all VOIs (supplementary Table 7).

Another limitation arises in the interpretation of ^11^C-UCB-J as a proxy for synaptic density. The extent to which SV2A binding reflects the entire pool of presynaptic vesicles, and to what extent the labeling of vesicles is dependent on the current physiological state of the synapse requires further validation [21, 38, 56]. Caution is also required when assuming that the number of presynaptic proteins SV2A is directly proportional to the number of synapses. Lastly, little is known about how psychotropic medication interferes with the binding of ^11^C-UCB-J. Except for an in vivo study with ketamine [57] and an in vitro study with lithium [58], which both had no effect on SV2A density in humans, it is not known how other antidepressants affect ^11^C-UCB-J binding. One theoretical concern is the effect of allosteric modulators of GABA-A, such as benzodiazepines, on SV2A distribution in GABAergic neurons. Post hoc analysis showed no intragroup differences between LLD patients taking benzodiazepines or not (supplementary table 8). Finally, whilst our power calculation was based on the only ^11^C-UCB-J study in depression (MDD/PTSD not LLD), we cannot exclude type II errors due to sample size and heterogeneity, for example, due to differences in medication use, symptom severity or the presence of anxiety, which we did not assess with formal rating scales.

In conclusion, we reproduced the finding of a trend in lower gray matter volume in regions implicated in late-life depression but found no evidence for altered synaptic density in those regions. In contrast to AD, lower gray matter volume in LLD seems not associated with lower synaptic density. Preserved synaptic density may suggest that the aging brain in LLD retains its capacity for neuroplasticity and remains amenable to therapeutic interventions. Future studies should replicate these preliminary findings and could investigate the clinical value of synaptic density as a differential diagnostic biomarker for LLD not associated with neurodegenerative diseases.

### Supplementary information

Supplementary figures

Supplementary tables

## Data Availability

All data that support the findings of this study are stored in a secured online research platform. Anonymized data are available upon reasonable request and approval by the local Ethics Committee.

## Code availability

PET image processing was conducted using the publicly available PSYPET 167 tool, version 1 which was developed by the author TVC. It is freely available to download from https://github.com/THOMVDC/PSYPET.
