# Supplementary material for: Preliminary evidence for preserved synaptic density in late-life depression
Source: Transl Psychiatry. 2024 Mar 14;14:145. doi: 10.1038/s41398-024-02837-8 (PMC10940592; doi:10.1038/s41398-024-02837-8)
Supplement: Supplementary file 1 [file 41398_2024_2837_MOESM1_ESM.docx]

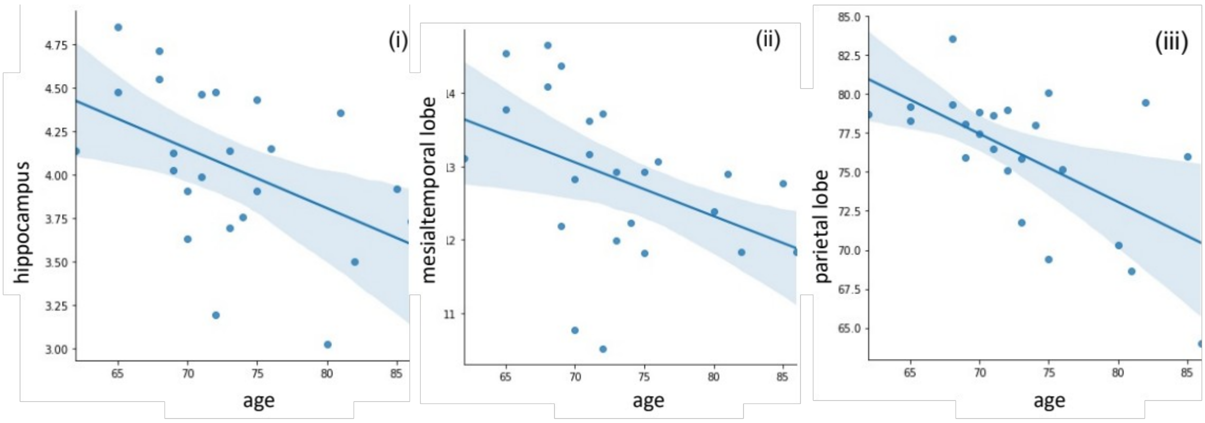

Supp figure 1: Correlation between age and gray matter volume and age in the (i) hippocampus, (ii) mesialtemporal and (iii) parietal lobe

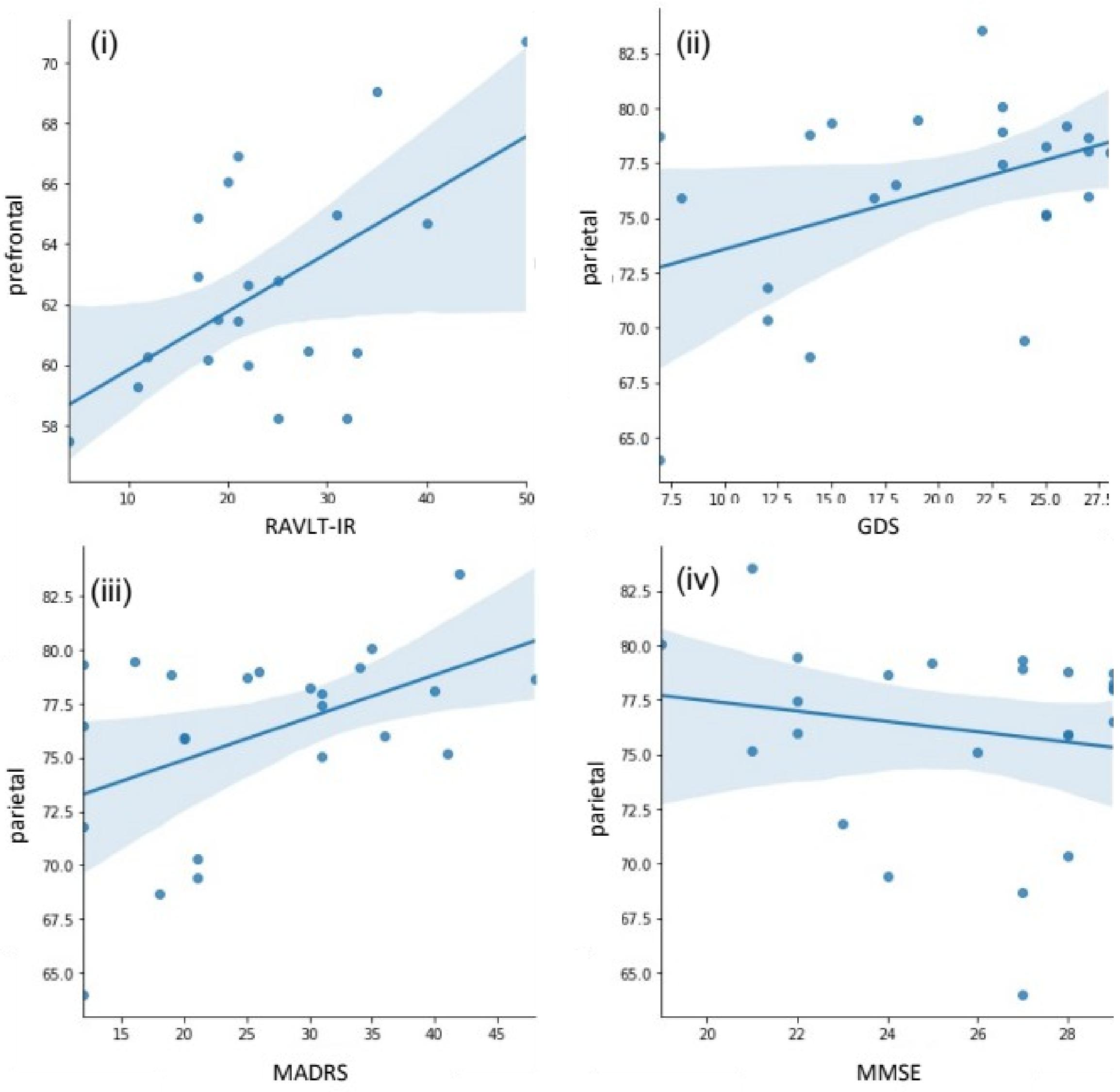

Supp figure 2: Correlation between prefrontal GMV and (i) RAVLT-IR, and between parietal GMV and (ii) GDS score, (iii) MADRS score and (iv) MMSE score

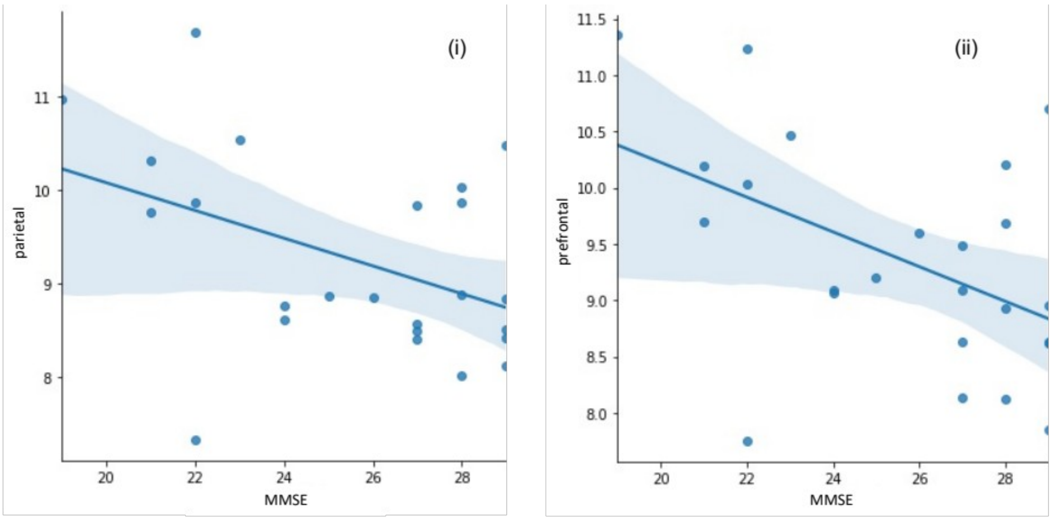

Supp figure 3: Correlation between MMSE and mean ^{11}C-UCB-J SUVR in the (i) parietal and (ii) prefrontal cortex

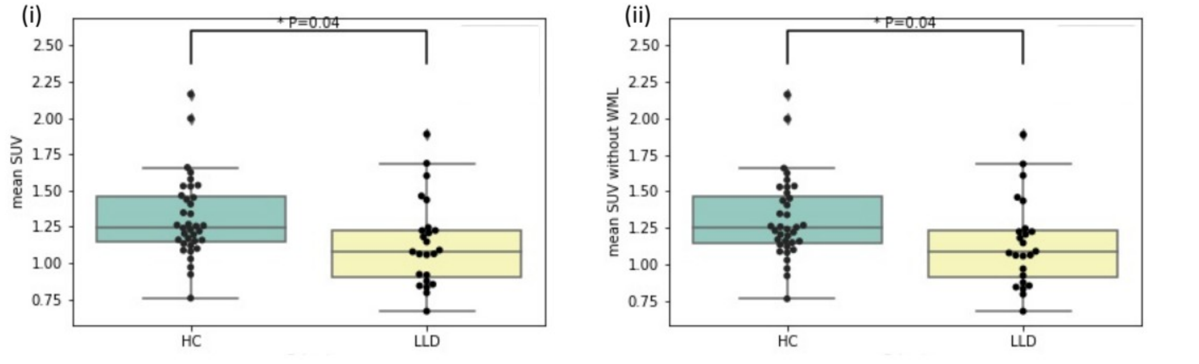

Supp figure 4 : Mean standardized uptake values (SUV) of ^{11}C-UCB-J in (i) the centrum semiovale, and in (ii) the centrum semiovale after exclusion of white matter lesions (WML)
