# Supplementary material for: Preliminary evidence for preserved synaptic density in late-life depression
Source: Transl Psychiatry. 2024 Mar 14;14:145. doi: 10.1038/s41398-024-02837-8 (PMC10940592; doi:10.1038/s41398-024-02837-8)
Supplement: Supplementary file 2 [file 41398_2024_2837_MOESM2_ESM.docx]

Supp. Table 1 Antidepressant medication intake of the late life depression (LLD) patients at inclusion of the study

	SSRI	SNRI	TCA	Mirtazapine	Trazodone
LLD1			1		1
LLD2					
LLD3	1			1	
LLD4	2		1	1	
LLD5	1				1
LLD6	2				
LLD7	1			1	
LLD8	1				
LLD9	1				
LLD10	1	1			1
LLD11	1			1	
LLD12	1				
LLD13		1			
LLD14		1		1	
LLD15			2	1	
LLD16	1				
LLD17			1		1
LLD18			1		
LLD19	1				
LLD20	1				
LLD21			1	1	1
LLD22	1			1	
LLD23		1		1	
LLD24	1		1		

Supp. Table 2A Volumetric values normalised by TIV (‰) reported in figure 2A

VOI	HC - median (IQR)	LLD - median (IQR)	P-value	F-statistic	DF
hippocampus	4.36 (0.47)	4.08 (0.69)	0.04	4.56	(1, 57)
mesotemporal	13.81 (1.20)	12.91 (1.49)	0.02	6.11	(1, 57)
lateral temporal	77.19 (4.61)	74.00 (4.35)	0.05	4.02	(1, 57)
prefrontal	65.32 (5.40)	61.62 (4.59)	0.02	6.33	(1, 57)
anterior cingulate	5.73 (1.12)	5.34 (0.36)	0.46	0.55	(1, 57)
parietal	76.45 (4.62)	77.72 (3.72)	0.13	2.30	(1, 57)

Supp. Table 2B SUVR values reported in figure 2B

VOI	HC - median (IQR)	LLD - median (IQR)	P-value	F-statistic	DF
hippocampus	5.76 (1.07)	5.68 (0.84)	0.74	0.11	(1, 58)
mesotemporal	5.37 (0.88)	5.62 (1.26)	0.05	3.98	(1, 58)
lateral temporal	8.67 (1.08)	8.73 (1.53)	0.22	1.54	(1, 58)
prefrontal	8.89 (1.10)	9.15 (1.44)	0.18	1.85	(1, 58)
anterior cingulate	7.92 (1.17)	8.55 (0.90)	0.09	2.99	(1, 58)
parietal	9.07 (0.77)	8.86 (1.41)	0.56	0.34	(1, 58)

Supp. Table 3 SUVR subgroup analysis based on depression onset

VOI	EOD - mean (sd)	LOD - mean (sd)	P-value	F-statistic	DF
hippocampus	5.68 (0.83)	5.82 (0.72)	0.68	0.18	(1, 22)
mesotemporal	5.72 (0.81)	6.16 (0.99)	0.25	1.39	(1, 22)
lateral temporal	8.93 (1.24)	9.27 (1.33)	0.53	0.40	(1, 22)
prefrontal	9.37 (1.00)	9.35 (1.04)	0.96	0.00	(1, 22)
anterior cingulate	8.56 (0.85)	8.57 (1.17)	0.97	0.00	(1, 22)
parietal	9.16 (1.03)	9.40 (1.16)	0.60	0.28	(1, 22)

Supp. Table 4 SUVR subgroup analysis based on psychotic symptoms

VOI	Non psychotic - mean (sd)	Psychotic - mean (sd)	P-value	F-statistic	DF
hippocampus	5.78 (0.54)	5.62 (1.16)	0.64	0.23	(1, 22)
mesotemporal	5.94 (0.92)	5.77 (0.86)	0.66	0.20	(1, 22)
lateral temporal	9.20 (1.34)	8.78 (1.09)	0.46	0.58	(1, 22)
prefrontal	9.46 (1.09)	9.17 (0.80)	0.51	0.44	(1, 22)
anterior cingulate	8.58 (1.03)	8.53 (0.84)	0.91	0.01	(1, 22)
parietal	9.35 (1.13)	9.05 (0.96)	0.52	0.42	(1, 22)

Supp. Table 5a SUVR subgroup analysis based on high severity late-life depression

VOI	HC - mean (sd)	High Severity - mean (sd)	P-value	F-statistic	DF
hippocampus	5.80 (0.82)	5.83 (0.78)	0.92	0.01	(1, 47)
mesotemporal	5.49 (0.66)	5.79 (0.81)	0.19	1.76	(1, 47)
lateral temporal	8.69 (1.02)	8.96 (1.10)	0.44	0.62	(1, 47)
prefrontal	9.00 (1.02)	9.49 (0.93)	0.14	2.28	(1, 47)
anterior cingulate	8.15 (0.88)	8.64 (0.75)	0.08	3.23	(1, 47)
parietal	9.09 (1.00)	9.20 (1.01)	0.74	0.11	(1, 47)

Supp. Table 5b SUVR subgroup analysis based on low severity late-life depression

VOI	HC - mean (sd)	Low severity - mean (sd)	P-value	F-statistic	DF
hippocampus	5.80 (0.82)	5.62 (0.79)	0.51	0.43	(1, 45)
mesotemporal	5.49 (0.66)	6.00 (1.00)	0.05	3.97	(1, 45)
lateral temporal	8.69 (1.02)	9.18 (1.46)	0.22	1.55	(1, 45)
prefrontal	9.00 (1.02)	9.22 (1.09)	0.55	0.36	(1, 45)
anterior cingulate	8.15 (0.88)	8.47 (1.18)	0.33	0.96	(1, 45)
parietal	9.09 (1.00)	9.31 (1.17)	0.56	0.35	(1, 45)

Supp. Table 6 SUVR non corrected for PVE

VOI	HC - mean (sd)	LLD - mean (sd)	P-value	F-statistic	DF
hippocampus	4.17 (0.47)	4.29 (0.48)	0.35	0.87	(1, 58)
mesotemporal	4.16 (0.45)	4.37 (0.59)	0.14	2.25	(1, 58)
lateral temporal	5.32 (0.58)	5.44 (0.72)	0.46	0.56	(1, 58)
prefrontal	4.95 (0.50)	5.03 (0.58)	0.53	0.39	(1, 58)
anterior cingulate	5.27 (0.57)	5.29 (0.59)	0.91	0.01	(1, 58)
parietal	5.04 (0.52)	5.19 (0.64)	0.33	0.98	(1, 58)

Supp. Table 7 SUVR PVC without WML extraction in the centrum semiovale

VOI	HC - mean (sd)	LLD - mean (sd)	P-value	F-statistic	DF
hippocampus	5.81 (0.83)	5.75 (0.79)	0.79	0.07	(1, 58)
mesotemporal	5.49 (0.66)	5.91 (0.95)	0.05	4.08	(1, 58)
lateral temporal	8.70 (1.02)	9.09 (1.33)	0.20	1.69	(1, 58)
prefrontal	9.01 (1.02)	9.40 (1.06)	0.16	2.04	(1, 58)
anterior cingulate	8.16 (0.88)	8.60 (1.02)	0.08	3.17	(1, 58)
parietal	9.10 (1.00)	9.28 (1.14)	0.51	0.44	(1, 58)

Supp. Table 8 SUVR subgroup analysis based on benzodiazepine intake

VOI	Naïve - mean (sd)	Non naïve - mean (sd)	P-value	F-statistic	DF
hippocampus	5.69 (0.96)	5.77 (0.59)	0.80	0.06	(1, 22)
mesotemporal	5.86 (0.81)	5.91 (0.99)	0.88	0.02	(1, 22)
lateral temporal	9.09 (1.23)	9.03 (1.33)	0.91	0.01	(1, 22)
prefrontal	9.39 (1.02)	9.34 (1.01)	0.91	0.01	(1, 22)
anterior cingulate	8.57 (0.90)	8.56 (1.05)	0.97	0.00	(1, 22)
parietal	9.28 (1.05)	9.22 (1.12)	0.91	0.01	(1, 22)

Supp. Table 9 SUVR in unilateral VOIs

VOI	HC - median (IQR)	LLD - median (IQR)	P-value	F-statistic	DF
hippocampus left	5.60 (1.13)	5.80 (1.20)	0.22	1.56	(1, 58)
hippocampus right	5.72 (1.03)	5.45 (0.99)	0.10	2.76	(1, 58)
mesotemporal left	5.33 (1.06)	5.71 (1.09)	0.03	4.95	(1, 58)
mesotemporal right	5.37 (0.87)	5.56 (1.44)	0.11	2.68	(1, 58)
lateral temporal left	8.62 (1.19)	8.75 (1.52)	0.23	1.45	(1, 58)
lateral temporal right	8.60 (1.08)	8.73 (1.52)	0.22	1.57	(1, 58)
prefrontal left	8.92 (1.09)	9.17 (1.46)	0.20	1.70	(1, 58)
prefrontal right	8.93 (1.15)	9.29 (1.32)	0.17	1.95	(1, 58)
anterior cingulate left	8.02 (1.23)	8.62 (1.03)	0.09	3.04	(1, 58)
anterior cingulate right	7.98 (1.05)	8.54 (0.80)	0.10	2.74	(1, 58)
parietal left	9.10 (0.70)	8.89 (1.30)	0.63	0.23	(1, 58)
parietal right	8.94 (0.82)	8.88 (1.56)	0.51	0.45	(1, 58)

Supp. Table 10a Mediation effects of synaptic density on the effect of late-life depression on gray matter volume

VOI	Average causal mediation effect	P-value
hippocampus	<0.01	0.97
mesotemporal	-0.07	0.44
lateral temporal	-0.09	0.53
prefrontal	0.06	0.84
anterior cingulate	-0.02	0.61
parietal	<0.01	0.93

Supp. Table 10b 11C-UCB-J mean SUVR correlations with gray matter volume in late-life depression

VOI	Pearson's r	P-value
hippocampus	-.02	0,91
mesotemporal	-.37	0,08
lateral temporal	-.29	0,19
prefrontal	.20	0,36
anterior cingulate	-.10	0,65
parietal	.26	0,23

Supp. Table 11 SUV and volume of the centrum semiovale before and after WML substraction

	SO before WML extraction		SO after WML extraction		Remaining volume (%)	Delta SUV
	SUV	Volume (mL)	SUV	Volume (mL)		
HC	1.31	1925.92	1.31	1906.75	.99	0
LLD	1.14	1903.04	1.14	1843.33	.97	0
HC+LLD	1.24	1916.77	1.24	1881.38	.98	0
